# A List-Ranking Framework Based on Linear and Non-Linear Fusion for Recommendation from Implicit Feedback

**DOI:** 10.3390/e24060778

**Published:** 2022-05-31

**Authors:** Buchen Wu, Jiwei Qin

**Affiliations:** 1 School of Information Science and Engineering, Xinjiang University, Urumqi 830046, China; buchen_wu@stu.xju.edu.cn; 2 Key Laboratory of Signal Detection and Processing, Xinjiang Uygur Autonomous Region, Xinjiang University, Urumqi 830046, China

**Keywords:** multilayer perceptrons, collaborative filtering, list ranking

## Abstract

Although most list-ranking frameworks are based on multilayer perceptrons (MLP), they still face limitations within the method itself in the field of recommender systems in two respects: (1) MLP suffer from overfitting when dealing with sparse vectors. At the same time, the model itself tends to learn in-depth features of user–item interaction behavior but ignores some low-rank and shallow information present in the matrix. (2) Existing ranking methods cannot effectively deal with the problem of ranking between items with the same rating value and the problem of inconsistent independence in reality. We propose a list ranking framework based on linear and non-linear fusion for recommendation from implicit feedback, named RBLF. First, the model uses dense vectors to represent users and items through one-hot encoding and embedding. Second, to jointly learn shallow and deep user–item interaction, we use the interaction grabbing layer to capture the user–item interaction behavior through dense vectors of users and items. Finally, RBLF uses the Bayesian collaborative ranking to better fit the characteristics of implicit feedback. Eventually, the experiments show that the performance of RBLF obtains a significant improvement.

## 1. Introduction

Many experiments have shown that deep neural networks (DNNs) are used in several fields because of their ability to capture complex and deeper information, including image segmentation [1], natural language processing [2,3], speech recognition [4], and recommendation systems [5,6,7]. Dailing Zhang et al. [8] designed deep learning-based frameworks that consist of both convolutional and recurrent neural networks to precisely identify human intentions in brain–computer interfaces. Kaixuan Chen et al. [9] proposed a semisupervised deep model for imbalanced activity recognition and pattern-balanced co-training for extracting and preserving the latent activity patterns to improve the robustness of co-training on imbalanced data. Minnan Luo et al. [4] exploited a novel semisupervised feature selection method through incorporating the exploration of the local structure to simultaneously learn the optimal graph. The primary assumption underlying the model is that the instances with similar labels should have a larger probability of being neighbors. With the large-scale application of neural networks in recommendation systems, it was found that neural networks that can fit most functions [10] are better than matrix factorization in extracting implicit user information. For instance, generative adversarial networks have gained increasing attention in the recommendation systems. CFGAN [11] and LARA [12] are pioneering methods to prove the potential of generative adversarial networks in recommendation systems. DeepCF [13] combines representation learning into a framework to overcome their disadvantages. A novel neural cooperative filtering (NCF) [14] framework based on deep learning directly learned user ratings of items and used MF and MLP to fit linear and nonlinear interactions. Ding, [15] et al. proposed the LMDB framework, which used modular functions to model the relevant attributes of each item and used discrete functions to describe the diversity attributes of the item set. Qiu, [16] et al. used content-rich domains to complement user representation and introduced user encoders and comment encoders to model the user’s behavior. Liu, [17] et al. found that directly fusing various types of side information into item embeddings brought less negative impact and better performance on the model.

Although all of these methods have achieved good results, they have different problems in user preference modeling and in terms of rating prediction. In terms of user preference modeling models, they either focus only on shallow information or only on in-depth information. Meanwhile, models based on MLP alone can easily fall into the overfitting problem and lose plenty of low-rank features. In terms of rating, there are many 0/1 ratings in implicit feedback, and the model has difficulty specifying the ranking order of these items with the same rating, thus limiting the model performance.

To solve these two problems, we propose a column label ordering architecture called RBLF that combines linear-nonlinear features with Bayesian coordination ranking for the first time. We set the potential features of user and item to different size dimensions to make the model more realistic. We explicitly fused user-embedding vectors and item-embedding vectors to learn the shallow linear interaction, feeding the obtained results into MLP to enhance the non-linearity of the model. The model fits the user–item feature relationship more comprehensively to guide the prediction method to better learn the user’s true preference attributes, thus giving more accurate rating prediction results. In the list ranking, we used Bayesian collaborative ranking, called deep-setrank [18], to better fit the features of implicit feedback in reality.

RBLF focuses on predicting the exact ranking of each item rather than the accuracy of specific scores. In practice, users mostly try to click the music ranked first in the list. Therefore, the ultimate goal of RBLF is list ranking. Furthermore, since there are far more implicit data (indirectly reflecting user preferences) in the real world than explicit feedback, this results in low cost of data collection. Our main contributions are as follows:

We propose a novel architecture to accommodate linear and non-linear interaction feature vector processes and design a neural network-based list-learning RBLF framework.

We use the deep-setrank list ranking and two other traditional list ranking algorithms. We compare these three ranking algorithms and conclude that our ranking algorithm is the best.

We explore the impact of the shallow and deep interaction behavior on feature grabbing layers.

We conduct many experiments on three datasets and show that RBLF greatly outperforms other recent algorithms.

## 2. Preparations

### 2.1. List-Ranking Methods

Many collaborative filtering technologies have been used in recommendation systems [19,20], and matrix factorization (MF) [21] has gained industrial recognition since it came out on top in the Netflix-Prize competition, creating many derivative models. Their prominent examples include a large number of enhancing MF, such as using them with biases [6] and extending them with the implicit parameters [22] to achieve universal feature modeling. Wang H, et al. [23] used feature matrices for capturing implicit user–item interactions [24]. Koren Y, et al. [25] used time-series matrix factorization to capture user preferences over time.

However, high-quality prediction accuracy and high-quality sorting recommendations are not strongly correlated [26,27]; thus, MF needs to be modified when applying it to the list sorting model. For example, Wu et al. [28] combined a learned ranking algorithm for lists with matrix factorization (MF) by modifying the loss function to linearly relate the observed scores of a given user–item matrix. Shi Y et al. [29] developed MF in the scenarios with binary relevance data.

There is little user–item interaction information because of the sparse rating matrix, and the performance of MF, which can be hindered by simply choosing the interaction function (inner product) [30], is poor in most cases. As the user rating matrix expands, simply linear combinations of the product of potential features cannot learn the users’ non-linear preferences. At the same time, they cannot effectively learn the implicit feature between users and items, which results in their poor ability to simulate implicit user feedback.

In recent years, DNNs, which can theoretically model any function, have been used in recommendation systems. Bai, et al. [31] created the NNCF model to capture localization information that traditional latent factor models cannot capture effectively, which integrates neighbor information as input into DNNs.

### 2.2. Implicit Feedback

Explicit feedback refers to numerical feedback with explicit rating criteria, such as the five-star rating system for MovieLens movies. Implicit feedback contains only positive and unobserved samples. Common implicit feedback includes click history, purchase history, like history, etc.

Given a recommendation problem, we suppose there is a series of users i and a series of items j. The recommendation task focused on in this paper requires both explicit feedback and implicit feedback from users on the items. Implicit feedback $y_{ij}$ is expressed as predicting whether there is an interaction and is defined as follows:

(1) $$y_{ij}=\left\{\begin{array}{l} 1\;\;user\;i\;has\;interaction\;on\;item\;j\\ 0\;\;otherwise\;\;\;\;\;\;\;\;\;\;\;\;\;\;\;\;\;\;\;\;\;\;\;\;\;\;\;\;\;\;\;\;\;\;\;\;\;\;\;\;\;\;\; \end{array}\right.$$

Here, $y_{ij}$ is 1, indicating that there is a click or browsing behavior between user i and item j. However, that does not mean that i truly likes j. Similarly, $y_{ij}$ is 0, which does not indicate that i is extremely averse to j, or that the user has not browsed and clicked on these items. Although the observed items at least reflect the user’s specific interests, the unchecked items may simply lose data.

In daily life, most users feedback is implicit, and the implicit feedback data will have a large number of identical items; this is a challenge for the list sorting model based on implicit feedback.

### 2.3. MLP

MLP is essentially a neural network. It aims to solve the nonlinear question that the single-layer perceptron cannot solve. The MLP model is defined as follows:

(2) $$\phi^{MLP}=\sigma \left(h^{T}\phi_{L}\left(wx+b\right)\right)$$

The structure of MLP is improved in two aspects from the single-layer perceptron structure:

(1) The hidden layer is added, which can have multiple layers to enhance the model’s expressive ability. However, it also increases the complexity of the model.
(2) To extend the activation function, although the activation function of the perceptron is simple, the processing capacity is limited. Thus, other activation functions are generally used in the neural network. By using different activation functions, the expression ability of the neural network is further enhanced.

For the activation function, sigmoid has limited the performance and caused a overfitting problem. Although tahn is applied more, it does not solve the above issues. The relu function is simple, has a fast-fitting speed, and will not cause oversaturation problems. We used the relu function.

(3) $$Relu\left(x\right)=\max\left(0,x\right)$$

## 3. The Experimental Model

**Figure 1:**
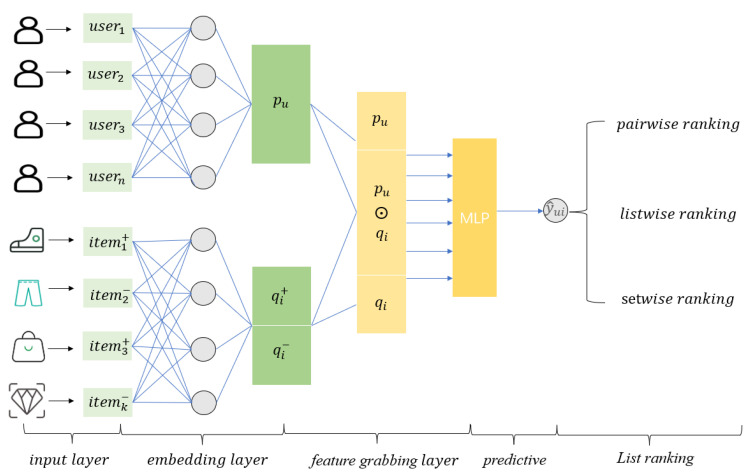
The architecture of RBLF.

**Figure 2:**
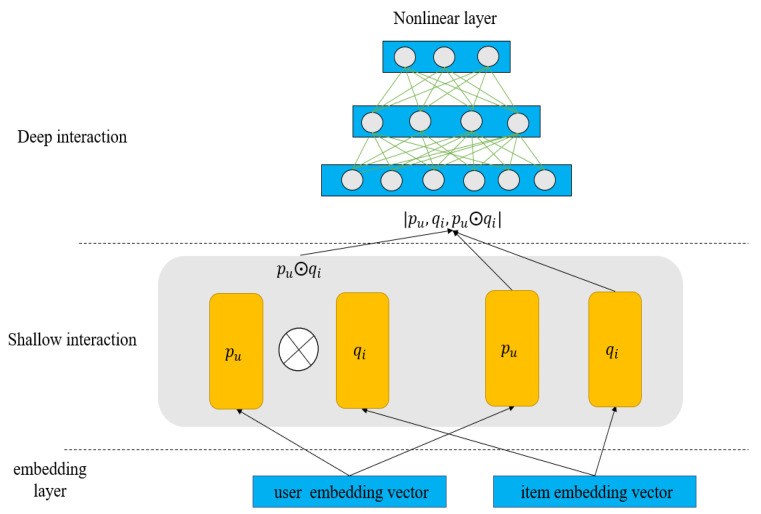
The architecture of interaction grabbing layer.

**Figure 3:**
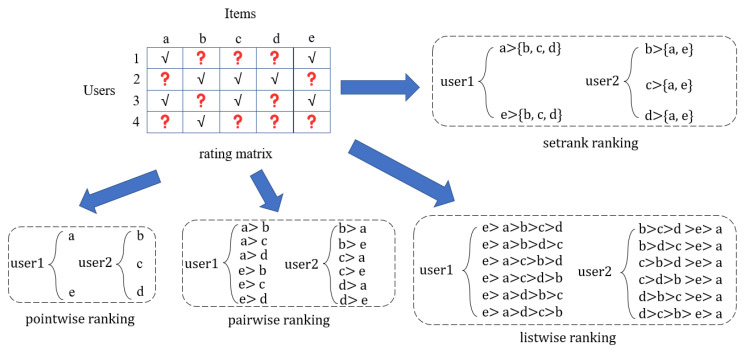
Comparison of different list ranking methods.

Figure 1 illustrates the overall framework of RBLF, Figure 2 shows the shallow-deep interaction grabbing layer structure of RBLF, and Figure 3 shows the idea of working with three different sorting algorithms. The second subsection details the interaction capture layer and describes how the layer works and the formula expression. In the third subsection, we describe in detail the formulas used by the four different sorting algorithms and how the results are sorted.

### 3.1. Input Layer and Embedding Layer

The input layer transforms the sparse user–item vector into one-hot encoding and feeds the result into the embedding layer.

The function of the embedding layer in RBLF is to transform the one-hot encoding of users and items into a low-dimensional space and represent them using a dense vector.

The embedding layer is defined as follows:

(4) $$p_{u}=f_{lookup}\left(u\right)$$

(5) $$q_{i}=f_{lookup}\left(i\right)$$

where $p_{u}$ and $q_{i}$ represent the embedding vectors of the user and item.

In reality, users and items are independent of each other, and they own a different number of latent features. Conversely, user preferences change over time, which leads to changing latent features. Still, items have relatively fixed attributes from the beginning of their creation; thus, the latent features of items do not change drastically [32]. We set different latent features. In the embedding layer, we use the list of $K^{+}$ positive items and $K^{-}$ negative items.$\;q_{i}^{+}$ and $q_{i}^{-}$ denote the positive and negative samples.

### 3.2. Shallow-Deep Interaction Grabbing Layer

We want to capture shallow linear user–item interactions and deep non-linear interactions in this layer.

First, the shallow interaction grabbing layer model carries the shallow user–item interaction behavior through explicitly fusing $p_{u}$ and $q_{i}$:

(6) $$X_{uk}=\left|\begin{array}{c} p_{u},q_{i},p_{u}⨀q_{i} \end{array}\right|$$

$p_{u}⨀q_{i}$ is the element-wise of two vectors. $\left|\begin{array}{c} p_{u},q_{i},p_{u}⨀q_{i} \end{array}\right|$ is the concatenation of these three vectors. By concatenating the $\left|\begin{array}{c} p_{u}⨀q_{i} \end{array}\right|$, the interaction can be better accommodated and prevent loss of feature [33]. We place $X_{uk}$ into the deep interaction grabbing layer.

The goal of the deep interaction grabbing layer is to learn deep and non-linear interaction behavior, and a standard MLP (multilayer perceptron) is used to learn the interaction latent features. Therefore, we can give the model nonlinear modeling capability rather than simply using the inner product multiplied element-by-element as MF (generalized matrix factorization) to describe the potential interaction characteristics. Since multilayer perceptron can simulate any function, we hope that this layer perceptron can better simulate the implicit preferences between user items. The model connects element-wise in series with multilayer perceptrons, which can be defined as follows:

(7) $$X_{uk}^{1}=\sigma \left(W_{n}^{1}X_{uk}+b_{n}^{1}\right)\;X_{uk}^{2}=\sigma \left(W_{n}^{2}X_{uk}^{1}+b_{n}^{2}\right)\;\ldots \;X_{uk}^{l}=\sigma \left(W_{n}^{l}X_{uk}^{l-1}+b_{n}^{l}\right)\;$$

$X_{ij}^{l}$ is the result of the lth-layer, $W_{n}^{l}$ $b_{n}^{l}\;$ is the weight matrix and bias.

### 3.3. Predictive Layer

#### 3.3.1. Pairwise Ranking

After we obtain the result of the interaction grabbing layer, we need the interaction grabbing layer to map the result to the probability ${\hat{y}}_{uk}$. Probability has two properties: the predicted probability is a nonnegative number. Softmax converts the prediction results from negative infinity to positive infinity through the promotion of the two classification functions sigmoid in multiclassification according to these two steps. The probability ${\hat{y}}_{uk}$ is formulated as follows:

(8) $${\hat{y}}_{uk}=softmax\left(X_{uk}^{l}\right)=\frac{e^{X_{uk}^{l}}}{\sum_{k=1}^{K}e^{X_{uk}^{l}}}$$

The pairwise RBLF is a special case of the listwise RBLF method. When the list is 2 (K = 2), the list-wise becomes the pairwise RBLF. The pairwise method models the relative ranking of double items to make predictions. Therefore, the pairwise method constructs the relationship between positive and negative examples. Then, the loss function we set through cross entropy is as follows:

(9) $$p\left(y,\hat{y}\right)=-\overset{U}{\underset{u=1}{\sum }}(\underset{i\in l_{u}^{+}}{\sum }\log{\hat{y}}_{ui}+\underset{j\in l_{u}^{-}}{\sum }\log(1-{\hat{y}}_{uj}))$$

$y_{ui}$ indicates the true probability, ${\hat{y}}_{ui}$ indicates the predicted probability, $l_{u}^{+}$ and $l_{u}^{-}$ represent interactive and noninteractive items by user u, respectively.

Compared with listwise RBLF, the pairwise RBLF is used to determine the relative order of two products and to integrate the results to obtain the final recommendation list, which emphasizes the short running time of the code.

Finally, we use regularization methods to avoid overfitting, and the regularization is defined as:

(10) $$P=p\left(y,\hat{y}\right)+\alpha (\overset{L}{\underset{l=1}{\sum }}{\begin{array}{c} \left\|W_{l}\right\| \end{array}}_{F}^{2}+\overset{U}{\underset{u=1}{\sum }}{\begin{array}{c} \left\|p_{u}\right\| \end{array}}_{F}^{2}+\overset{I}{\underset{i=1}{\sum }}{\begin{array}{c} \left\|q_{i}\right\| \end{array}}_{F}^{2})$$

where ${\begin{array}{c} \left\|\cdot \right\| \end{array}}_{F}^{2}$ represents the Fibonacci-norm and α is the regularization coefficient set in the experiment.

#### 3.3.2. Listwise Ranking

As in method pairwise RBLF, the listwise RBLF also needs the interaction-grabbing layer to map the result to the probability ${\hat{y}}_{uk}$. Unlike method pairwise RBLF, the listwise RBLF learns the sample features of an ordered list instead of learning an ordered pair. The probability of items is defined as:

(11) $$p\left(S\left(i_{1},i_{2},\ldots ,i_{K}\right)\right)=\underset{i\in l_{u}^{+}}{\prod }{\hat{y}}_{ui}\underset{j\in l_{u}^{-}}{\prod }1-{\hat{y}}_{uj}$$

where $S\left(i_{1},i_{2},\ldots ,i_{K}\right)$ denotes the set of all items in list $l_{u}$ and K denotes the number of items in the list $l_{u}$. Then, the model simulates the distribution between the true list and the predicted list by cross entropy. The listwise ranking with regularization is defined as follows:

(12) $$p\left(y,\hat{y}\right)=-\overset{U}{\underset{u=1}{\sum }}\left(\underset{i\in l_{u}^{+}}{\sum }\log{\hat{y}}_{ui}+\underset{j\in l_{u}^{-}}{\sum }\log(1-{\hat{y}}_{uj})\right)+\;\alpha (\overset{L}{\underset{l=1}{\sum }}{\begin{array}{c} \left\|W_{l}\right\| \end{array}}_{F}^{2}+\overset{U}{\underset{u=1}{\sum }}{\begin{array}{c} \left\|p_{u}\right\| \end{array}}_{F}^{2}+\overset{I}{\underset{i=1}{\sum }}{\begin{array}{c} \left\|q_{i}\right\| \end{array}}_{F}^{2})$$

#### 3.3.3. Deep-Setrank

Considering that each user’s rating process can be approximated as independent of each other and not influenced by other users, we first assume that each user’s rating results are independent. Then, for each user, its preference for the positive sample can be considered higher than that of the unobserved sample. Thus, we can compare each positive sample of users with the set of unobserved samples and assume that the probability of a user liking a positive sample is greater than the probability of liking the set of unobserved samples. We can then maximize the likelihood probability values of these comparisons to solve the problem. Compared to the pairwise assumption, the setrank assumption avoids the problem of inconsistent independence by relaxing the independence requirement. The Bayesian posterior probability of the setrank preference structure can be given as:

(13) $$p\left({>}_{total}|\Theta \right)=\overset{U}{\underset{u=1}{\prod }}p\left({>}_{u}|\Theta \right)=\overset{U}{\underset{u=1}{\prod }}\underset{k\in l_{u}}{\prod }p\left(l_{u}^{+}>l_{u}^{-}|\Theta \right)$$

where ${>}_{total}\;$ is the preference structure of all users, ${>}_{u}$ is a random variable representing the preference structure of the representation user u, and Θ is the parameter to be learned in the scoring modeling section. $p\left(l_{u}^{+}>l_{u}^{-}|\Theta \right)$ represents the probability that a positive sample $l_{u}^{+}$ is better than the preference structure of the set consisting of some unobserved samples $\;l_{u}^{-}$. This probability can be equivalently considered as the probability that this positive sample ranks first in the list consisting of this positive sample and all unobserved samples, while the order between unobserved samples or within positive samples is not to be considered; thus, there is no problem in sorting items with the same rating by the listwise method.

As shown in Figure 3, the preferred items for user 1 are $\left\{a,e\right\}$ and the unobserved items are $\left\{b,c,d\right\}$. We only need to express that $a>\left\{b,c,d\right\}$ and $e>\left\{b,c,d\right\}$ without sorting between items $\left\{a,e\right\}$ and $\left\{b,c,d\right\}$. In the embedding layer, we use the list of one positive items, and K−1 negative items.

Our method only cares about the sorting probability when a positive sample is ranked first; thus, introducing the listwise method’s list order-based probability modeling formula to calculate the probability can be simplified as:

(14) $$p_{s,1}\left(l_{u}^{+}>l_{u}^{-}|\Theta \right)=\frac{\emptyset \left(s_{l_{u}^{+}}\right)}{\sum_{a=1}^{K}\emptyset \left(s_{a}\right)}$$

where $p_{s,1}\left(l_{u}^{+}>l_{u}^{-}|\Theta \right)$ denotes the probability that item $l_{u}^{+}$ is ranked first, and $s_{a}\;$ is the rating of item a.

The complete probabilistic modeling form of the setrank method as:

(15) $$p\left({>}_{total}|\Theta \right)=\overset{U}{\underset{u=1}{\sum }}\underset{k\in l_{u}}{\sum }-log\frac{\emptyset \left({\hat{y}}_{ui}\right)}{\emptyset \left({\hat{y}}_{ui}\right)+\sum_{j\in l_{u}^{-}}^{K-1}\emptyset \left({\hat{y}}_{uj}\right)}$$

Since we do not sort the set of unobserved items or the set of positive sample items internally, we use the sigmoid function to map the results of the interaction-grabbing layer into probabilities:

(16) $$log\emptyset ({\hat{y}}_{uk})=sigmoid\left(X_{uk}^{l}\right)=\frac{1}{1+e^{X_{uk}^{l}}}$$

By means of maximizing the posterior probabilities, the final optimization objective function can be given as:

(17) $$P=\overset{U}{\underset{u=1}{\prod }}\underset{k\in l_{u}}{\prod }p\left({>}_{total}|\Theta \right)+\alpha (\overset{L}{\underset{l=1}{\sum }}{\begin{array}{c} \left\|W_{l}\right\| \end{array}}_{F}^{2}+\overset{U}{\underset{u=1}{\sum }}{\begin{array}{c} \left\|p_{u}\right\| \end{array}}_{F}^{2}+\overset{I}{\underset{i=1}{\sum }}{\begin{array}{c} \left\|q_{i}\right\| \end{array}}_{F}^{2})$$

## 4. The Experiment Evaluation

In this section, we introduce the dataset, the experimental evaluation index and the algorithm to compare with our experiment. Our experiment aims to answer four key questions

1. RQ1: How does RBLF perform compared to the currently popular list-sorting algorithms?
2. RQ2: What are the effects of shallow and deep interaction methods in the feature capture module on RBLF?
3. RQ3: What are the effects of different list ranking methods in the predictive layer on RBLF?
4. RQ4: How does it impact the effect of different hyperparameters of the model?

### 4.1. Datasets, Evaluation Metrics and Compared Models

As shown in Table 1, we evaluated our model on several public datasets, including MovieLens—100 k, MovieLens—1 M, and Yahoo. We randomly selected 80% of the scores for training the data for each dataset.

In addition, we used two popular accuracy metrics, the HR@N and the NDCG@N (N denotes that the RBLF generate the number of the top-n items).

The larger the value of HR and NDCG, the better the model’s performance. The HR@N score is defined as:

(18) $$HR@N=\frac{\sum_{u=1}^{\#users}\frac{hits}{N}}{\#users}$$

where #users are the total users whose items in the test set appear.

NDCG@N is defined as follows:

(19) $$DCG\left(b,N\right)=\overset{b}{\underset{i=1}{\sum }}r_{i}+\overset{N}{\underset{i=b+1}{\sum }}\frac{r_{i}}{log_{b}i}$$

(20) $$NDCG=\frac{DCG}{iDCG}$$

$r_{i}$ indicates whether the item ranked i is preferred by the user. $r_{i}=1$ indicates that the user likes the product; $r_{i}=0$ indicates that the user does not like the product; b is the free parameter, which is generally set to 2; N is the number of the top-n items from RBLF. DCG was normalized to obtain NDCG.

We used the compared methods as shown below:

The ItemKNN model considers the evaluation bias after the calculation is completed and obtains the k most similar items.

BPR [34] is a widely used Bayesian-sorting algorithm.

ListRank-MF [26] uses a learning-sorting algorithm and matrix factorization to improve performance while maintaining low complexity.

Neural cooperative filtering (NCF) [14] directly uses a combination of the DNN and matrix factorization, thereby alleviating the problem of DNN overfitting and ignoring low-rank information.

The DeepCF [13] model is the deep matrix decomposition model (DMF) and gives its own solution to NCF’s problem. It uses matching learning, which combines the advantages of the two methods, and effectively avoids the shortcomings of the two methods.

The DeepRank [32] model is built on natural language processing capabilities and is currently one of the best sorting algorithms.

### 4.2. Performance Evaluation (RQ1)

The model is compared with the benchmark in Table 2. The best marks are highlighted in bold.

**Figure 4:**
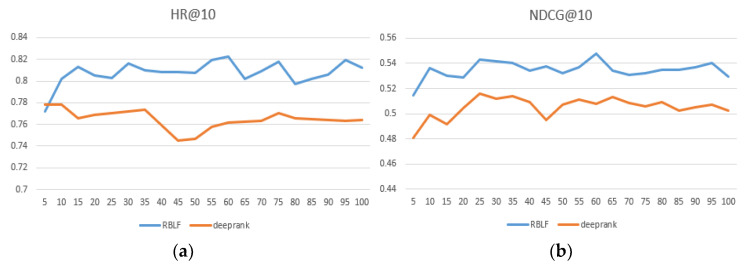
(**a**) HR@10 comparison of RBLF and DeepRank in MovieLens—100 K; (**b**) NDCG@10 comparison of RBLF and DeepRank in MovieLens—100 K.

**Figure 5:**
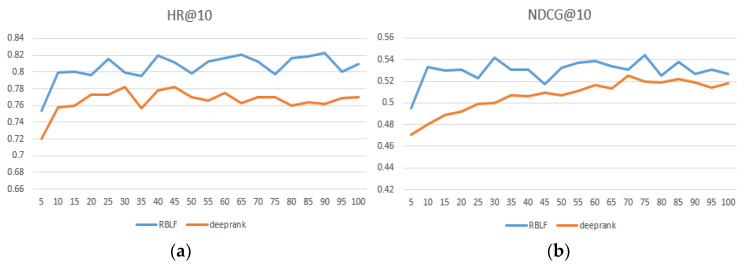
(**a**) HR@10 comparison of RBLF and DeepRank in MovieLens—1 M; (**b**) NDCG@10 comparison of RBLF and DeepRank in MovieLens—1 M.

**Figure 6:**
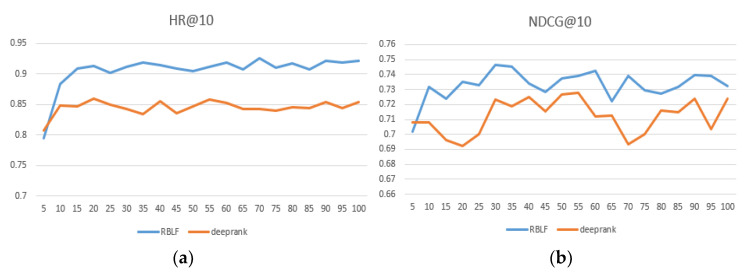
(**a**) HR@10 comparison of RBLF and DeepRank in YaHoo Movie; (**b**) NDCG@10 comparison of RBLF and DeepRank in YaHoo Movie.

We compared the performance of RBLF and the adaptability of the model when facing different datasets in Table 2 and the best performing numbers are in bold. At the same time, we intuitively compared the DeepRank model, which is the closest to our model and records results within each epoch at 100. As seen in Figure 4, Figure 5 and Figure 6, the x-axis represents the epoch and the y-axis represents the results.

As seen in Table 2, our proposed method achieves excellent ranking performance and has considerable advantages on each dataset. We believe that it is precisely because our model better simulates the user’s preferences that the performance is ahead of the performance of the comparison algorithm. In addition, RBLF consistently outperforms the DeepRank model on the three datasets and increases by 6.3%, 7.1%, and 4.5%, respectively. (According to the paper and our experimental data, the specific values of the hyperparameters when DeepRank achieves the best performance are: the length of the list K = 15, the user and item dimension sizes of embedding $d_{u},d_{u}=\left\{16,\;8\right\}$, and the depth of MLP L = 4.) Figure 4, Figure 5 and Figure 6 show that the RBLF and DeepRank epoch are between 0–100, including the values of HR@10 and NDCG@10. In the figure, we can see more clearly that the performance of RBLF is better at each epoch, and at the same time, it avoids DeepRank due to the problem of mid-term performance degradation.

On the sparsest dataset Yahoo! Movie, RBLF is also superior to other methods, indicating that the idea of combining RBLF in our model simulates the invisible preferences of users, ensuring high performance and high flexibility of the model. Since BPR and ListRank-MF are simply linear interactions, they perform relatively poorly on all datasets, although they also model invisible preferences. The DeepRank model uses the MLP model, which omits some low-rank information and some simple user characteristics in the user–item matrix. As a result, although their performance is high, they are still inferior to RBLF. Although the NCF model uses MLP and MF, our model is a cascade fusion, which allows the model to better integrate these two algorithms, and thus, our model performance is even better. The DeepCF method is concerned with the point-by-point method and ignores the paired ranking information. Our model captures the user’s characteristics from the user’s paired item. This result in our model is more powerful than theirs in predicting the performance of personalized rankings.

### 4.3. Ablation Experiments of Shallow and Deep Interaction Methods (RQ2)

As shown in Table 3, √ represents whether the feature capture layer includes this module. We did three types of experiments in total and recorded the performance changes when the model only has a shallow interaction grabbing layer, only a deep interaction grabbing layer, and a complete shallow deep interaction grabbing module. The performance of the RBLF model decreases by 1.5% when the shallow interaction-grabbing layer is lost. In comparison, the overall performance of the model reduces by 3.4% when the multilayer perceptron for grabbing the deep interaction is lost. This proves that the model for capturing shallow interaction has less impact on the overall performance than MLP, and the nonlinear module can better model the user’s preference.

### 4.4. Different List Ranking Methods (RQ3)

The results for different list ranking methods are shown in Table 4. Deep-setrank outperforms the pairwise ranking and listwise ranking on the two datasets and increases by 10% and 1.8%, respectively.

The reason Deep-setrank performance is substantially better than pairwise ranking is that pairwise methods typically model the preference structure in implicit feedback based on an item pair consisting of a positive feedback item and an unobserved item. This approach is prone to the problem of inconsistent independence in assumptions and implementation. Pairwise ranking attempts to maximize the probability of pairwise comparisons between positive and unobserved simples. This work requires the strict assumption that two items have independent pairwise preferences as the basis for constructing the loss function. Therefore, the independence between preference pairs cannot be guaranteed, which affects the optimization results of the pairwise loss function. Only the order of the two documents is considered, and the position of the documents in the search list is not, resulting in a less-than-optimal final ranking.

The reason Deep-setrank outperforms listwise ranking is that the list method is implemented by defining a probabilistic relationship between the preference sizes on the list of items. For the list method, items with the same rating value cannot be handled efficiently, especially because there is no explicit graded rating in the implicit feedback but 0/1 rating, which can lead to a large number of items with the same rating. In contrast, Deep-setrank does not sort the set of unobserved items or the set of positive sample items internally but only ensures that each positive sample is larger than the set of unobserved samples. Therefore, Deep-setrank models the implicit feedback data more realistically than listwise ranking.

### 4.5. Different Hyperparameters of the Model (RQ4)

The effect of the length of the list is shown in Table 5. First, as the list length K increases, the model performance increases. After K = 5, the performance is not significantly improved. After K = 10, the performance of the model begins to decrease as K increases. This is because our model is more complex, and the meaningless increase in the length of the list affects the final performance. At the same time, the longer list length inevitably leads to a substantial increase in the running time. Therefore, we finally take K as 5, the optimal parameter after combining time and performance.

**Figure 7:**
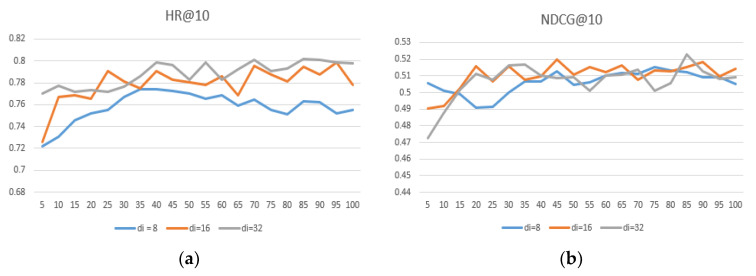
(**a**) $d_{u}$ = 8, comparisons of HR@10 under different $d_{i}$ in MovieLens—100 K; (**b**) $d_{u}$ = 8, comparisons of NDCG@10 under different $d_{i}$ in MovieLens—1 M.

**Figure 8:**
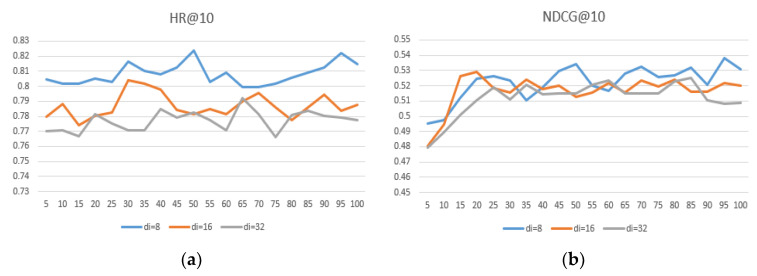
(**a**) $d_{u}$ = 16, comparison of HR@10 under different $d_{i}$ in MovieLens—100 K; (**b**) $d_{u}$ = 16, comparison of NDCG@10 under different $d_{i}$ in MovieLens—1 M.

**Figure 9:**
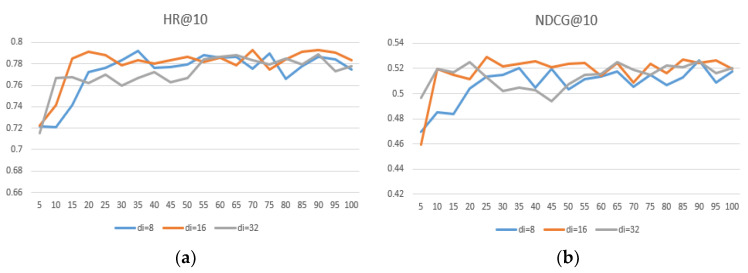
(**a**) $d_{u}$ = 32, comparison of HR@10 under different $d_{i}$ in MovieLens—100 K; (**b**) $d_{u}$ = 32, comparison of NDCG@10 under different $d_{i}$ in MovieLens—1 M.

The embedding size is important for the representation of the project. We conducted experiments to determine the impact of embeddings of different dimensional sizes on the performance of the model. We set the user and item embedding dimension size $d_{u}=\left\{8,\;16,\;32\right\}$, e $d_{i}=\left\{8,\;16,\;32\right\}$, and the results are shown in Table 6. Figure 7, Figure 8 and Figure 9 show that the user embedding remains unchanged in MovieLens—100 K and the impact of changing the size of the item embedding, where the x-axis represents the epoch and the y-axis represents the results.

After conducting many experiments, we can conclude the following: First, when the user–item embedding dimensions are different, the performance is better than that of the user and the item with the same embedding dimension. Second, if you want to achieve better results, neither user embedding nor item embedding can take 32 because excessive dimensions.

Third, the dimension of user embedding is preferably smaller than the dimension of item embedding. Finally, we conclude that when $d_{u}$ = 8 and $d_{i}$ = 16, the model’s performance can reach its highest and performs much better on different datasets, proving that the generalization ability is well.

In the new experiment, we set the size of MLP to [8], [16,8], [32,16,8], [64,32,16,8] and [128,64,32,16,8], and the results are shown in Table 7.

In the beginning, as the number of layers of MLP increases, the performance of the model also improves because the deep neural network is similar to the structure of the shallow neural network when the number of layers is too small, and the model does not have sufficient fitting capabilities. Therefore, as L increases, the fitting ability of the deep neural network also increases, which drives the improvement of the model’s performance. After L = 4, the performance decreases instead because the model adds a shallow interaction-grabbing layer, limiting the MLP model’s depth.

In this section, we compare the performance and time cost of three list ranking methods. Table 8 shows the training time of the three models on the MovieLens—100 K and Movielens—1 M datasets.

The time cost of the pairwise-ranking model is far superior to that of those models, although the performance of pairwise-ranking is the worst. As for listwise-ranking and deep-setrank, they take about the same amount of time, but the performance of deep-setrank is better than the listwise-ranking. Thus, we suggest that if you care about the time cost, you should choose pairwise-ranking, and if you prefer higher performance, you should choose deep-setrank.

## 5. Conclusions and Future Work

This paper proposes a list-ranking framework based on linear and non-linear fusion for recommendation (RBLF). The model addresses the problem in that current list-sorting recommender systems always fail to capture the full range of user–item interaction information by adding a shallow–deep interaction-grabbing layer, thus improving the model performance.

In the future, we want to optimize the way to obtain the potential vectors of users and items. As the theory of graph neural networks matures, the potential vectors obtained by graph neural networks are much richer than one-hot coding and embedding. In addition, multimodal auxiliary information can make the features more adequate, such as using the item’s image and the user’s comment to optimize the feature vector.

## Figures and Tables

**Table 1:** Statistics of the datasets.

Datasets	Users	Items	Rating	Sparsity
ML-100 K	943	1682	100,000	93.63%
ML-1 M	6040	3952	1,000,000	95.81%
Yahoo	7642	11,915	211,231	99.77%

**Table 2:** Comparison results in different datasets.

Dataset	Metrics	HR@5	HR@10	NDCG@5	NDCG@10
MovieLens—100 K	ItemKNN	0.482	0.498	0.339	0.362
	BPR	0.536	0.668	0.371	0.411
	List-rank MF	0.511	0.653	0.355	0.399
	NCF	0.627	0.721	0.432	0.472
	DMF	0.653	0.750	0.431	0.487
	DeepRank	0.778	0.768	0.521	0.511
	**RBLF**	**0.812**	**0.826**	**0.533**	**0.541**
MovieLens—1 M	ItemKNN	0.452	0.478	0.226	0.279
	BPR	0.516	0.696	0.351	0.413
	List-rank MF	0.488	0.637	0.344	0.389
	NCF	0.607	0.711	0.381	0.447
	DMF	0.693	0.725	0.437	0.445
	DeepRank	0.749	0.761	0.493	0.499
	**RBLF**	**0.789**	**0.819**	**0.531**	**0.529**
Yahoo	ItemKNN	0.482	0.498	0.339	0.362
	BPR	0.641	0.796	0.571	0.641
	List-rank MF	0.618	0.737	0.556	0.608
	NCF	0.785	0.821	0.648	0.694
	DMF	0.787	0.825	0.657	0.703
	DeepRank	0.853	0.868	0.695	0.713
	**RBLF**	**0.928**	**0.917**	**0.722**	**0.741**

**Table 3:** Comparative results of ablation experiments.

Dataset	Module		Evaluation Indicators	
	Shallow	Deep	HR@10	NDCG@10
100 K		√	0.803	0.518
	√		0.787	0.509
	√	√	0.815	0.521
1 M		√	0.795	0.517
	√		0.774	0.515
	√	√	0.813	0.519
Yahoo		√	0.881	0.736
	√		0.870	0.711
	√	√	0.895	0.740

**Table 4:** Comparison results for different list ranking methods.

List—Methods	MovieLens—100 K		MovieLens—1 M	
	hr@10	ndcg@10	hr@10	ndcg@10
pairwise ranking	0.7407	0.4756	0.7429	0.4940
listwise ranking	0.8080	0.5271	0.7937	0.5390
Deep-set rank	0.8234	0.5408	0.8191	0.5291

**Table 5:** Comparison results for the different lengths of the list.

K	MovieLens—100 K		MovieLens—1 M		Yahoo! Movie	
	hr@10	ndcg@10	hr@10	ndcg@10	hr@10	ndcg@10
2	0.7440	0.4849	0.7494	0.4867	0.8515	0.6706
5	0.8235	0.5480	0.8185	0.5486	0.8991	0.7453
10	0.8189	0.5282	0.7993	0.5319	0.8941	0.7354
15	0.8218	0.5329	0.8033	0.5366	0.8990	0.7312

**Table 6:** Comparison results in the different dimension sizes of embedding.

$d_{u}$	$d_{i}$	MovieLens—100 K		MovieLens—1 M	
		hr@10	ndcg@10	hr@10	ndcg@10
8	8	0.7467	0.5003	0.7676	0.5113
8	16	0.8167	0.5348	0.8071	0.5274
8	32	0.8006	0.5329	0.7991	0.5148
16	8	0.8145	0.5231	0.8058	0.5228
16	16	0.8021	0.5217	0.7964	0.5199
16	32	0.7826	0.5232	0.7637	0.5190
32	8	0.7607	0.5156	0.7629	0.5140
32	16	0.7544	0.5074	0.7553	0.4999
32	32	0.7588	0.0.5095	0.7487	0.5029

**Table 7:** Comparison results for different numbers of layers for MLP.

L	MovieLens—100 K		MovieLens—1 M	
	hr@10	ndcg@10	hr@10	ndcg@10
1	0.7504	0.5149	0.7691	0.4731
2	0.7916	0.5289	0.7667	0.4768
3	0.8181	0.5300	0.7996	0.4986
4	0.8234	0.5408	0.8191	0.5291
5	0.8049	0.5326	0.7885	0.4861

**Table 8:** Time cost for different list ranking methods.

	MovieLens—100 K	Movielens—1 M
Pairwise-ranking	2 m 12 s	14 m 49 s
Listwise-ranking	5 m 5 s	38 m 20 s
Deep-setrank	5 m 1 s	36 m 22 s

## Data Availability

We evaluated our algorithm on three datasets: MovieLens—100 k, MovieLens—1 M, and Yahoo. https://grouplens.org/datasets/MovieLens/100k/ (accessed on 15 October 2020); https://grouplens.org/datasets/MovieLens/1m/http://webscope.sandbox.yahoo.com/catalog.php?datatype=r&did=75 (accessed on 16 October 2020).
